# Dynamic weight bearing analysis is effective for evaluation of tendinopathy using a customized corridor with multi-directional force sensors in a rat model

**DOI:** 10.1038/s41598-017-07812-6

**Published:** 2017-08-18

**Authors:** Po-Ting Wu, Chieh-Hsiang Hsu, Fong-Chin Su, I-Ming Jou, Shih-Yao Chen, Chao-Liang Wu, Wei-Ren Su, Li-Chieh Kuo

**Affiliations:** 1 Department of Orthopedics, College of Medicine, National Cheng Kung University, Tainan, Taiwan; 2 Department of Orthopedics, National Cheng Kung University Hospital, College of Medicine, National Cheng Kung University, Tainan, Taiwan; 3 Department of Biomedical Engineering, National Cheng Kung University, Tainan, Taiwan; 4 Medical Device Innovation Center, National Cheng Kung University, Tainan, Taiwan; 5 Department of Orthopedics, E-DA Hospital, Kaohsiung, Taiwan; 6 Department of Internal Medicine, College of Medicine, National Cheng Kung University, Tainan, Taiwan; 7 Department of Biochemistry and Molecular Biology, College of Medicine, National Cheng Kung University, Tainan, Taiwan; 8 Department of Occupational Therapy, College of Medicine, National Cheng Kung University, Tainan, Taiwan

## Abstract

Few studies discuss kinetic changes in tendinopathy models. We propose a customized corridor to evaluate dynamic weight bearing (DWB) and shearing forces. Sixty rats were randomly given ultrasound-assisted collagenase injections (Collagenase rats) or needle punctures (Control rats) in their left Achilles tendons, and then evaluated 1, 4, and 8 weeks later. The Collagenase rats always had significantly (p < 0.001) higher histopathological and ultrasound feature scores than did the Controls, significantly lower DWB values in the injured than in the right hindlimbs, and compensatorily higher (p < 0.05) DWB values in the contralateral than in the left forelimbs. The injured hindlimbs had lower outward shearing force 1 and 4 weeks later, and higher (p < 0.05) push-off shearing force 8 weeks later, than did the contralateral hindlimbs. Injured Control rat hindlimbs had lower DWB values than did the contralateral only at *week 1*. The Collagenase rats had only lower static weight bearing ratios (SWBRs) values than did the Controls at *week 1* (p < 0.05). Our customized corridor showed changes in DWB compatible with histopathological and ultrasound feature changes in the rat tendinopathy model. The hindlimb SWBRs did not correspond with any tendinopathic changes.

## Introduction

Tendinopathy is a chronic painful tendon disorder that is common in athletes and the sedentary^1^. Its pathophysiological mechanism is still unclear; thus, a valid animal model is needed to evaluate its etiology, molecular mechanism, and potential treatments. There are several types of tendinopathy animal models. Intratendinous collagenase injection is a common induction method that yields consistent tendon damage compared with mechanical-overload models. It is also less labor-intensive and less expensive to develop^1, 2^. Animal models, however, can only approximate human tendinopathy^1^, because it cannot reproduce the course of the human version of the disease.

Ultrasound is a real-time noninvasive examination with a growing number of applications for monitoring and diagnosing tendinopathy. Common ultrasound findings, such as increased tendon thickness, tendon calcification, neovascularization, and focal hypoechoic change, are correlated with the severity of tendinopathy^3–5^. Therefore, ultrasound is not only a reliable clinical assessment tool^6^, but is also considered essential for evaluating animal models^1^. However, few studies report the dynamic changes in ultrasound features after a collagenase injection in a rat tendinopathy model.

Pain assessment is important when evaluating animal models and the therapeutic efficacy of different interventions. Many methods have been developed to investigate pain in small animals, including spontaneous locomotor activities^7^, static weight bearing ratio^8^, mechanical sensitivity (von Frey test)^9^, thermal sensitivity^10^, vocalization^11^, and gait analysis^12–14^. Because of advances in technology, gait analysis has become increasingly popular in rodent models^14^. Compared with histopathological and ultrasound examinations, gait analysis has the advantages of being non-invasive and of having a functional perspective. Fu *et al*.^13^ in 2009 reported that motion analysis can be used to measure the painful responses associated with collagenase-induced tendinopathy rat models. However, kinetic changes in tendinopathy models have seldom been discussed. Therefore, we propose a customized corridor composed of a chain of load cells and one digital camera to evaluate the kinetic parameters (dynamic weight bearing (DWB) force and shearing force) during a gait analysis. Our first hypothesis was that collagenase injected into a rat’s Achilles tendon would induce histopathological changes, ultrasound features, and DWB changes that approximate human tendinopathy. Our second was that, because of the pain, the collagenase-treated hindlimb would present less DWB than would the contralateral hindlimb, and the contralateral forelimb would present compensatorily more DWB than would the right forelimb.

## Results

### Histopathological grading

**Figure 1:**
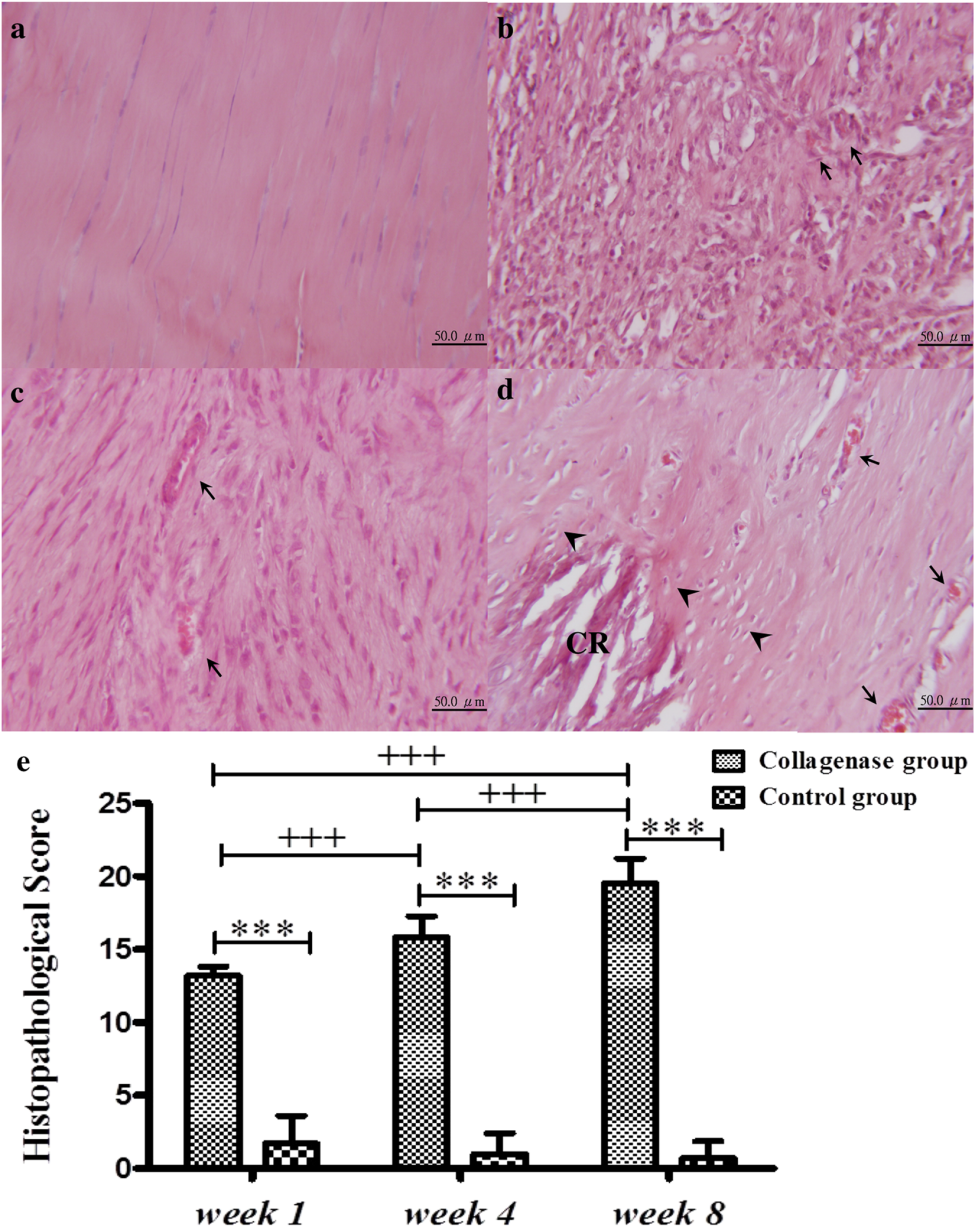
Hematoxylin and eosin staining and histopathological scores of Achilles tendons after an intratendinous injection of collagenase or only needle punctures. Control (**a**) and injured tendons at *weeks 1* (**b**), *4* (**c**), and *8* (**d**). The summary of histopathological score (**e**). CR, calcified deposits; arrowhead, chondrocyte-like cells; arrow, vascular structure. The histopathological scores: *compared with the Control group; ^+^compared within the Collagenase group; ^*^/^+^p < 0.05; ^**^/^++^p < 0.01; ^***/+++^p < 0.001.

The histopathological scores in the Collagenase group increased significantly over time: 13.2 ± 0.6 at *week 1*; 15.8 ± 1.5 at *week 4*, and 19.5 ± 1.7 at *week 8*. The difference between each time point was significant (all p < 0.05) (Fig. 1e). Calcific deposits, the histological hallmarks of calcified tendinopathy, were first observed at *week 4* and were present in all samples at *week 8* (Fig. 1d). Chondrocyte-like cells (Fig. 1d), another characteristic of tendinopathy, usually surrounded the calcific deposits and were first present at *week 4*, as reported elsewhere^15^. Other characteristics of tendinopathy and tendinitis, including neovascularization, hypercellularity, and loss of matrix organization, were also present from *week 1* (Fig. 1b–d). The tendon had not healed by *week 8*. These characteristics were not present in the Sham Control (hereafter Control) group throughout the study. Tenocytes were well aligned within longitudinally arranged collagen fibrils in the Control group (Fig. 1a). The histopathological scores in the injured tendons were significantly (all p < 0.01) higher than in the Control tendons throughout the study (Fig. 1e).

### US features scores

**Figure 2:**
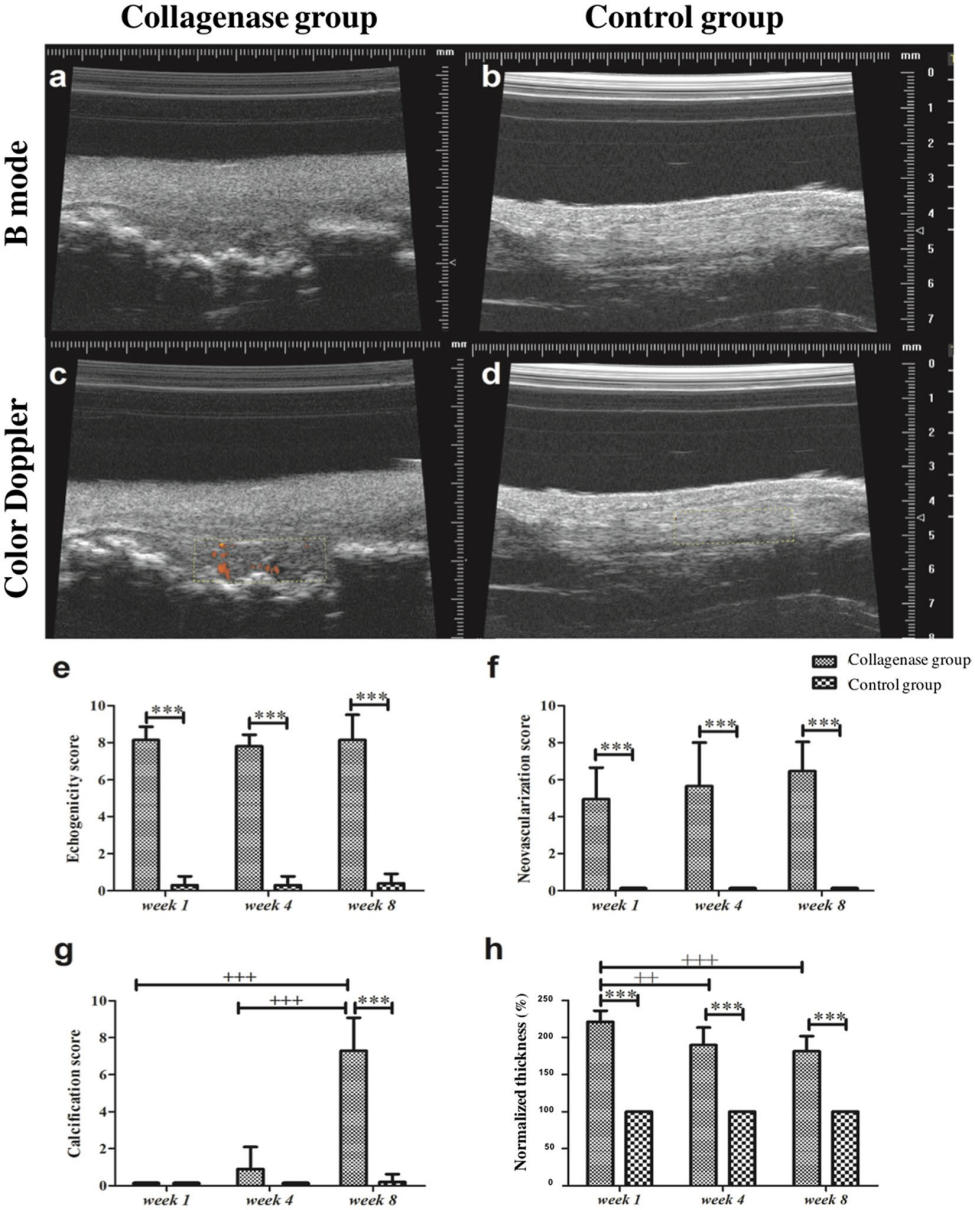
The ultrasound images and ultrasound features scores. At *week 8*, the collagenase group showed increased tendon thickness, diffuse hypoechoic changes, multiple calcification (**a**), and abundant neovascularization (**c**). In the Control group, such ultrasound features were seldom visible (**b** and **d**). The summary scores of US features, including echogenicity (**e**), neovascularization (**f**), calcification (**g**), and normalized thickness (**h**). *Compared with the control group; ^+^compared within the collagenase group; ^*/+^p < 0.05; ^**/++^p < 0.01; ^***/+++^p < 0.001.

The echogenicity score in the collagenase group was persistently high from *week 1* to *week 8* (Fig. 2a,e). The neovascularization score tended to increase nonsignificantly with healing time (Fig. 2c,f). The calcification score, which was significantly (p < 0.001) higher at *week 8*, increased as post-injury healing progressed, (Fig. 2a,c,g). These ultrasound features of hypoechoic changes, neovascularization, and calcification were seldom found in the Control tendons throughout the study (Fig. 2b,d,e–g). Furthermore, these ultrasound scores, except for calcification scores at *weeks 1* and *4*, were significantly (all p < 0.001) higher in the injured tendons than in the control tendons throughout the study (Fig. 2e–g). The percentage of normalized tendon thickness was significantly (p < 0.01) higher at *week 1* in the Collagenase group, and was also significantly (p < 0.001) higher compared with the Control group at each time point (Fig. 2h).

### Validity and reliability tests of the customized corridor

The results of the validity test of each force plate in all three axes are shown in Supplementary Table 1. The R^2^ values of the regression analyses were larger than 0.9984 (p < 0.001). The ICC values for repeatability were 1, which indicated the high consistency and reliability of data acquisition. The recorded values of each force plate were corrected based on the linear regression results and used for subsequent kinetic analyses.

### Kinetic parameters and static weight bearing ratio

**Figure 3:**
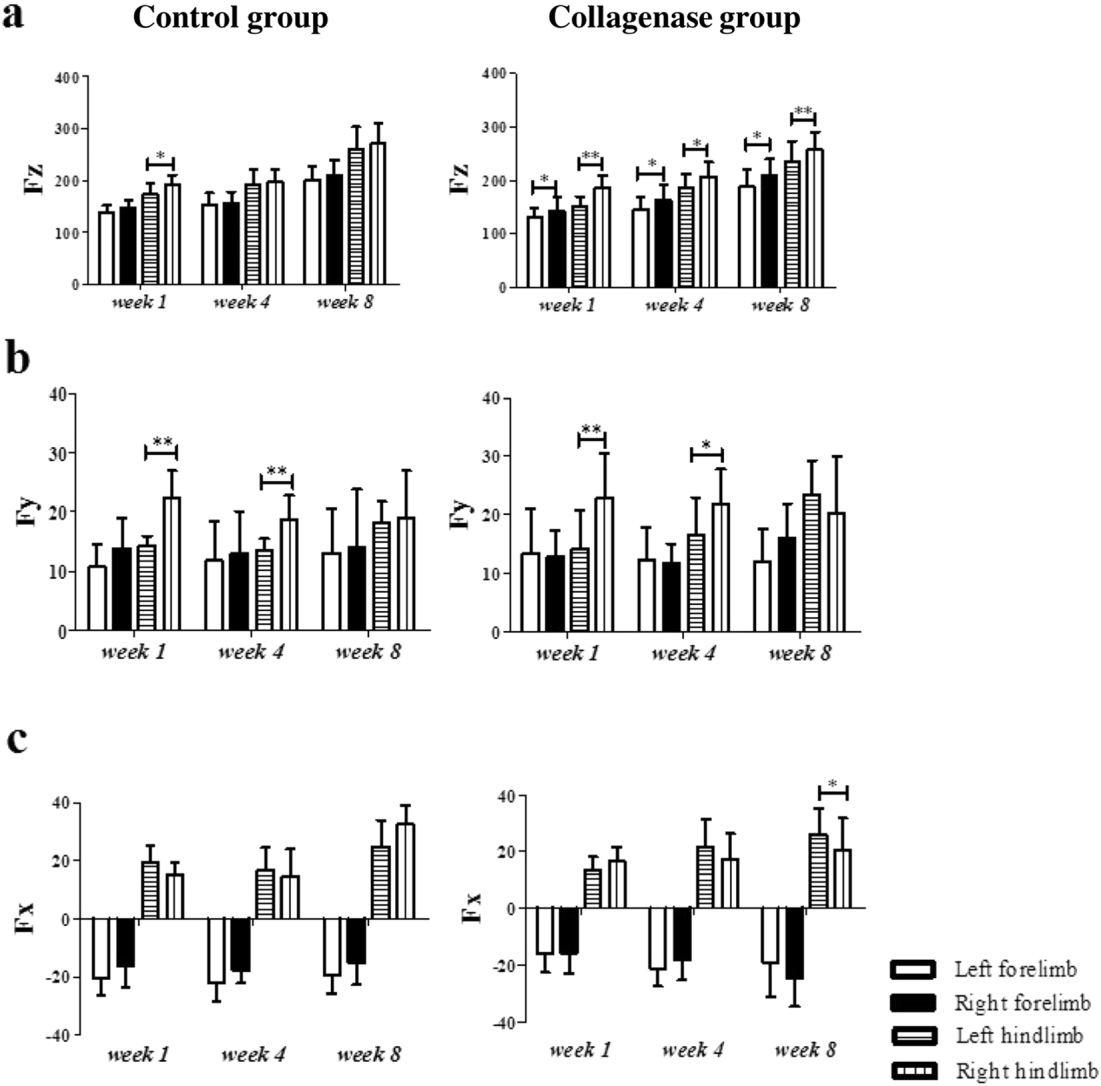
The force values of the three axes in the rat gait analysis. The left hindlimbs were injected with collagenase (Collagenase group) or given only needle punctures (Sham Control group). The right hindlimbs were given no treatment (internal controls). (**a**) Fz, vertical direction; (**b**) Fy, inward (negative)/outward (positive) direction (value); (**c**) Fz, forward (negative)/backward (positive) direction (value). *Compared with contralateral forelimbs and hindlimbs; *p < 0.05; **p < 0.01.

**Figure 4:**
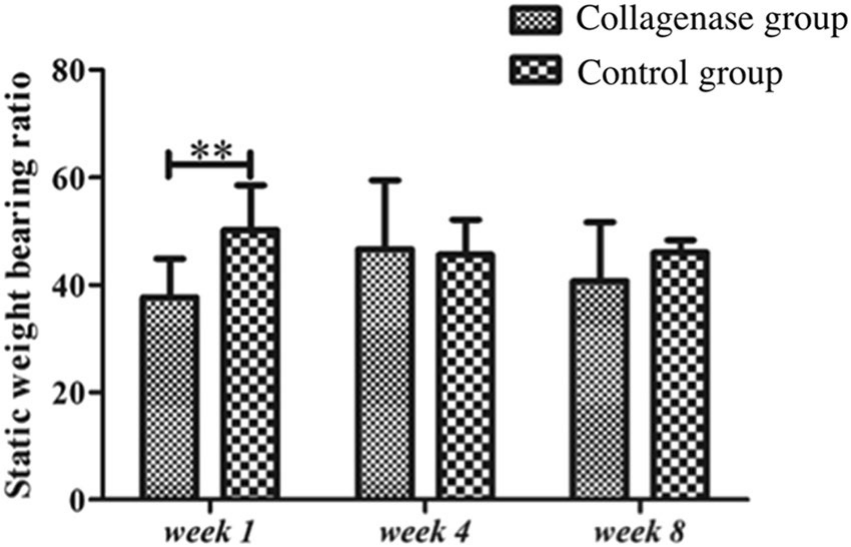
The static weight bearing ratio of the injured hindlimbs. *Compared with the Shane Controls; **p < 0.01.

In the Collagenase group, the injured (left) hindlimbs had significantly (all p < 0.05) lower DWB values than did the contralateral (right) hindlimbs throughout the study period. Moreover, the contralateral forelimbs always had compensatorily and significantly (all p < 0.05) higher DWB values than did the ipsilateral (left) forelimbs (Fig. 3a). Control group rats did not undergo such kinetic changes; however, DWB was significantly (p < 0.05) lower in the left than in the right hindlimbs at *week 1* (Fig. 3a). Additionally, the Fy value of each limb was in an outward direction, and the Fx value was in the landing direction in the forelimbs and push-off direction in the hindlimbs. In the Collagenase group, the Fy value of the injured hindlimbs was significantly (p < 0.05) lower than that of the contralateral hindlimbs at *weeks 1* and *4*, but not at *week 8* (Fig. 3b). The Fx value of the injured hindlimbs was significantly (p < 0.05) higher than that of the contralateral hindlimbs at *week 8* (Fig. 3c). In the Control group, the Fy value was also significantly (p < 0.05) lower in the injured hindlimbs than that in the contralateral hindlimbs at *weeks 1* and *4* (Fig. 3b). The Fx value showed no significant difference throughout the study period. The static weight bearing ratio (SWBR) was significantly (p < 0.01) lower in the Collagenase group than in the Control group only at *week 1*(Fig. 4).

## Discussion

This is the first study to evaluate the DWB and shear forces during walking in a rat tendinopathy model, and to validate the changes in kinetic features with other tendinopathic features. We found that the ultrasound-assisted collagenase injection into rat Achilles tendons is valid, and that the Collagenase-group rats had correspondingly higher histopathological and ultrasound feature scores, and significantly lower DWB scores than did the Control-group rats. Up to *week 8*, the injured tendons still showed the histopathological hallmark of tendinopathy: a failure of the tendon to heal. The Collagenase group showed significantly lower DWB values in the injured hindlimbs than in the right hindlimbs, and compensatorily higher DWB values in the contralateral forelimbs than in the left forelimbs at all time points. The injured hindlimbs also had lower outward shearing force (Fy) values at *weeks 1* and *4*, and higher push-off shearing force (Fx) values at *week 8* than did the contralateral hindlimbs. In the Control group, the injured hindlimbs had lower DWB values than did the contralateral hindlimbs only at *week 1*, and lower Fy values at *weeks 1* and *4*. The SWBR values in the Collagenase group were lower than those in the Control group only at *week 1*.

Histologically, tendinopathy models should represent a defective healing response with the following characteristics: hypercellularity, neovascularity, extracellular matrix (ECM) degradation, rounding of cell nuclei, and acquisition of chondrocyte phenotypes^1^. After the index procedure in our study, the histopathological score in the Collagenase group significantly increased with the healing time, and was significantly higher than the score in the Control group, in which the score showed no significant differences during healing. Moreover, the above histopathological features of tendinopathy were all observed in our collagenase-induced tendinopathy model at *week 8*. Our findings are consistent with other studies^1, 16^ which reported that the collagenase-induced tendinopathy rat model presents a persistent defect without healing from *week 8*.

Ultrasound provided structural information, e.g., thickness, calcification, echogenicity, alignment, and tearing, of in patients with tendinopathy^6, 17^. Furthermore, US can clearly reveal neovascularization using color Dopplor tests^18^. Blood vessels are seldom visible inside a healthy endotendon. Although neovascularization in ultrasound imaging is not always revealed in every tendinopathic tendon, it is still regarded as a hallmark of tendinopathy^19^. We found that all ultrasound features had higher scores in the Collagenase group than in the Control group, except for calcification at *weeks 1* and *4*. Tendon calcification, which was first visible at *week 4*, corresponded with ectopic calcification in the histopathological changes: thus, it continued to be visible at *week 8*. This is earlier than the time reported by Liu *et al*. (*week 12*)^15^. A possible reason for this is the different target tendon used in these two studies. However, as the injured tendons in the Collagenase group healed, their thickness decreased. This is a common criticism of the collagenase-induced tendinopathy model: it induces acute and severe inflammation in the early stage, and then the injured tendons begin to repair themselves. Finally, the injured tendons did not heal, which validated the tendinopathy model.

Gait-analys is identifies the most important parameters for assessing musculo skeletal disease models, and spatiotemporal, kinematic, and kinetic analyses are three common methods of obtaining such data. Spatiotemporal analysis has been widely applied to assess pain-related behaviors^14, 20–22^ and tendinopathy/tendinitis animal models^13^. The SWBR is one common parameter in kinetic gait analyses. It is measured using an incapacitance tester to assess the pain-related behavior in osteoarthritis^23^ and tendinopathy models^7^. Surprisingly, we found that SWBRs did not reflect the pain behavior in the collagenase-induced tendinopathy rat model. The Collagenase group showed less weight bearing ratio in the injured hindlimbs than that did Control group only at *week 1*, because of the acute inflammation. At *week 8*, when the model matured and represented failed tendon healing and corresponding ultrasound features, the Collagenase group still showed no significant difference in the weight bearing ratio of the hindlimbs compared with the Control group. Yoshida *et al*.^7^ also reported no decrease in the SWBR in the overloading tendinopathy model using a rodent treadmill. A possible reason for this is that the rats assumed a standing position by leaning against the incapacitance tester apparatus when SWBR was measured. The static testing position thus facilitated the adaptation, and possibly masked the pain-related behaviors. Our review of the English literature revealed no reports of a decreased SWBR in a collagenase-induced tendinopathy model. However, the DWB or ground reaction force is another common parameter of kinetic gait analyses in animal models using commercial systems^24, 25^ or customized platforms^26–28^. TekScan^24^ and Bioseb^25^, two major commercial systems, provide, in grams or in mm^2^ (area), simultaneous weight-bearing data from four limbs, and spatiotemporal information, e.g., different gait-phase durations and stride lengths. The limitations of such systems are their high cost and lack of shearing force data. For be spoke systems, the three-axes force changes of the targeted forelimb in a rotator cuff tendon-injury model (surgical detachment) using customized platforms^26–28^. Their limitation is that only data from the injured forelimb were provided because there were only two force plates. Our system used four force plates to acquire weight-bearing data from four limbs during the gait cycles. However, more force plates would have made it difficult for the rats to have a “satisfactory” walk. Finally, our customized corridor clearly showed the lower values in the injured hindlimbs and compensatorily higher values in the contralateral forelimbs throughout the study. These kinetic changes of the injured hindlimbs are compatible with the histopathological and ultrasound feature findings for the injured Achilles tendons. Moreover, the injured hindlimbs had lower outward shearing force (Fy) values at *weeks 1* and *4*, and higher push-off shearing force (Fx) values at *week 8* than did the contralateral hindlimbs, which might be an adaptation response. Interestingly, the Control group had lower DWB values in the left hindlimbs (given needle punctures only) than in the contralateral hindlimbs at *week 1*, and Fy values at *weeks 1* and *4*. Although there were no significant differences in histopathological scores or ultrasound feature scores in the Control group during the healing, needle punctures might have induced a transient pain reaction at *week 1* and a subtle change in Fy values. DWB values while walking are thus more representative and reliable than are SWBR values for assessing pain behaviors in a rat Achilles tendinopathy model. Our customized corridor system is easy to setup and less expensive than are commercial systems, and it detected the DWB and shearing force distribution in all four limbs.

This study has some limitations. First, we did not directly evaluate the expression of the pain-related sensory peptides in the injured tendon to correlate them with pain-related behaviors. However, Lui *et al*.^15^ clearly showed, during healing, the significantly higher expression of substance P and calcitonin gene-related peptide in the Collagenase groups than in the Control groups. However, our aim was to evaluate the kinetic changes, one method of pain assessment in a collagenase-induced rat Achilles tendinopathy model. Therefore, we did not repeat a similar analysis of sensory peptides. Second, we did not evaluate spatiotemporal features such as gait cycle and velocity, other kinetic parameters such as ankle- and knee-moment changes, and kinematic parameters that can theoretically be measured using our system. Fu *et al*.^13^ reported that changes in spatiotemporal parameters are associated with pain in a tendinopathy rat model. However, studies^13, 29^ have reported imprecise measurements of knee and ankle angles because of large skin movements during changes in position which makes it difficult to assess joint-moments and joint-kinematic changes. We thus focused on the evaluation of three-axes of DWB. Third, we did not control the gait velocity or the interstride variability that affects gait DWB and shearing force. Because it had been reported^30^ that freedom of movement reduced the possibility of stress-induced analgesia, we allowed the rats to walk freely in the corridor, as has been done in other studies^25, 31, 32^. Finally, our results showed that this method was adequate for measuring the significant differences in DWB between injured and healthy tendons. Little is known about rodent DWB^14^; thus, additional studies and more advanced methods of gait analysis are necessary to better understand rodent gait patterns in pain-related assessments.

Our customized corridor revealed the DWB and changes in shearing force in the four limbs of the rats while they were walking. Injecting collagenase into rat Achilles tendons induced significantly less DWB in the injured tendons than in the contralateral hindlimbs, and compensatorily higher DWB in the contralateral forelimbs until *week 8*. These kinetic changes are compatible with changes in histopathological and ultrasound features. However, the SWBR of the hindlimbs did not correspond with the tendinopathic changes. Overall, the results of this study show that an ultrasound-assisted collagenase injection into a rat Achilles tendon is a valid model of Achilles tendon tendinopathy.

## Materials and Methods

### Ethics Statement

All experimental rats were purchased from the Animal Center at National Cheng Kung University, and the subsequent experiments were done in accordance with the relevant guidelines and regulations approved by the Institutional Animal Care and Use Committee of National Cheng Kung University (protocol number: 102064).

### Animal Model

Sixty male Sprague-Dawley rats (8 weeks old; weight, 250–300 g) were randomly assigned to one of two groups: Collagenase and Sham Control (n = 30 each). To induce tendinopathy in the Collagenase group, the left Achilles tendons were intratendinously injected, using a 29-gauge needle, with 20 µL (0.015 mg/µL in 0.9% saline, or 0.3 mg) of bacterial collagenase I (Sigma-Aldrich, St. Louis, MO, USA)^16^. In the Sham Control group, the left Achilles tendons were given only needle punctures. Each right Achilles tendon received no treatment as the internal control. Every injection and needle puncture procedure was assisted using an ultrasound (Vevo 770; Visual Sonics, Toronto, Canada) 55-MHz linear transducer for high-resolution images. After the index procedure, rats in each group were randomly assigned to one of three post-induction timepoints: *week 1*, *week 4*, and *week 8* (n = 10). Ultrasound examinations and kinetic analysis were used to analyze the rats, were euthanized for histopathological examinations on the post-injection days indicated in their group names: *weeks 1*, *4*, and *8*.

### Ultrasound guidance procedures

Before the ultrasound-assisted injections or needle punctures and ultrasound examinations, all of the rats were anesthetized with isoflurane, and the posterior skin hair of both hindlimbs was removed using an electric razor. The rat was placed prone on a flat, heated pad to maintain its body temperature. The hindlimbs were secured, and a sterile coupling gel was loaded to cover the Achilles tendon after it had been disinfected. Two-dimensional real-time B-mode scanning was used to visualize the Achilles tendon, and the scanning window was centered on the tendon, with a 10.0 × 10.0-mm field of view. The ultrasound transducer was held in a hands-free stand positioned 4.5 mm above the center of the tendon. The axis of the linear transducer was aligned along the long axis of the Achilles tendon. Using real-time ultrasound assisted, the needle was placed parallel to the long axis of the ultrasound transducer and the injection was confirmed to be intratendinous. As the collagenase solution slowly infiltrated into the target, the ultrasound images clearly showed the inflation of the tendon.

### Scoring of US features

After the index procedure, and before they were euthanized, the ultrasound features of Achilles tendons were evaluated *in vivo* at *weeks 1*, *4*, and *8* by an experienced orthopedic surgeon (IMJ) who was blinded to the study protocol. The levels of echogenicity were scored from 0 to 10 based as previously described^25^: (hypoechoic area, 0 = no change, 10 = change throughout tendon), neovascularization (visible vessels, 0 = none, 10 = ≥10 vessels), and calcifications (number of calcifications, 0 = none, 10 = ≥10 calcifications). The thickness of the Achilles tendons was also measured at the center of the tendon and expressed as a percentage increase after each had been normalized with each right Achilles tendon.

### Histopathological grade

The left Achilles tendons, from the muscle-tendon junction to the calcaneal insertion site, were harvested from each rat. Each specimen was fixed in fresh 4% paraformaldehyde for 16–24 h at 4 °C, and then subsequently dehydrated, paraffin-embedded, and longitudinally sectioned. Sequential 4-µM sections were stained with hematoxylin and eosin (H&E). We used a semiquantitative method to score each factor on a four-point system: 0 = normal, 1 = slightly abnormal, 2 = moderately abnormal, and 3 = markedly abnormal. The following parameters were assessed as described elswhere^29^: fiber structure, fiber arrangement, roundness of the nuclei, regional variations in cellularity, increased vascularity, decreased collagen stainability, fibrosis or hyalinization, and calcification characteristics. The maximum total score for each specimen was 24. Using a light microscope, one blinded examiner assessed the extent of tendinosis.

### Customized corridor design

**Figure 5:**
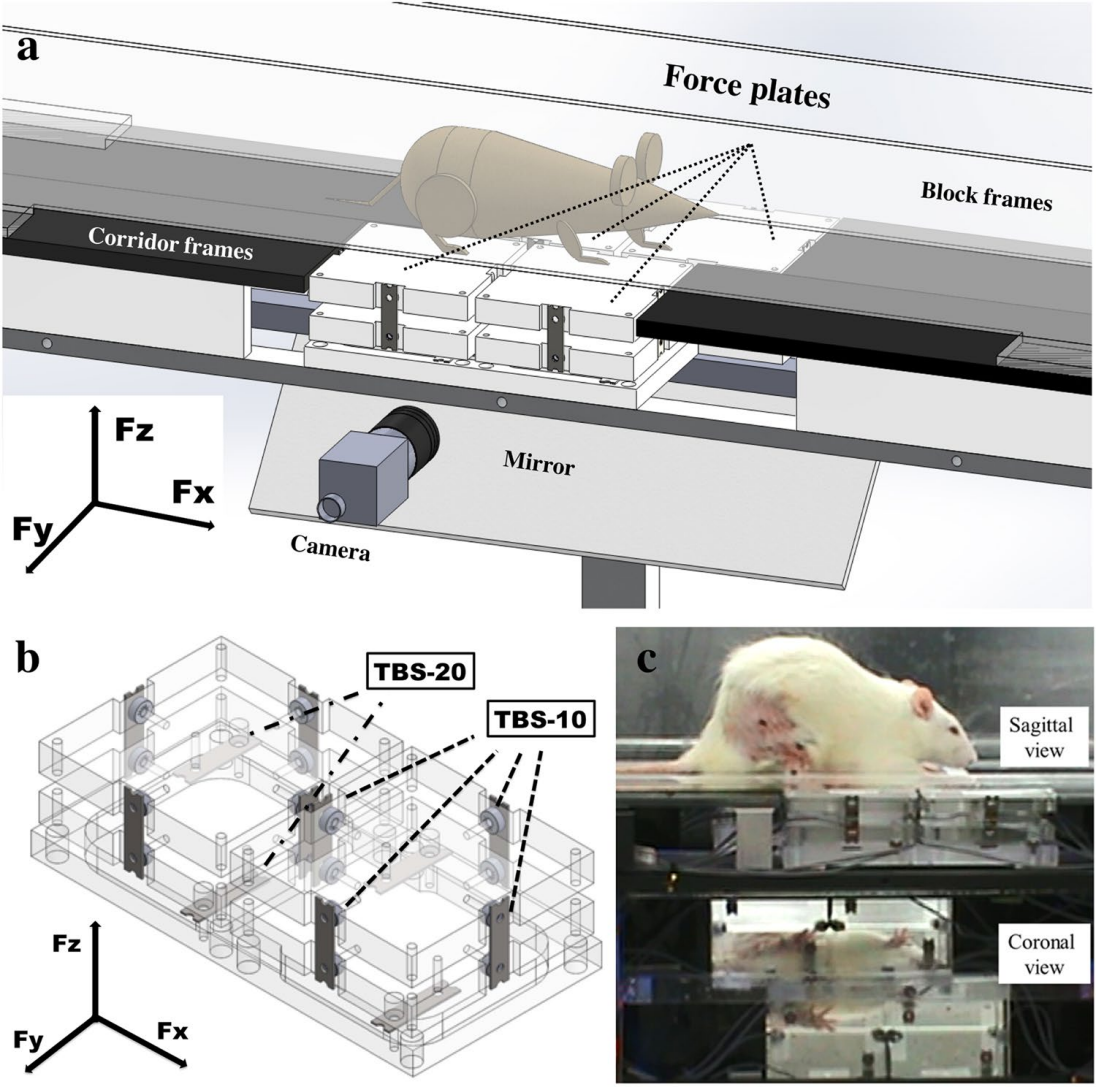
The system consisted of four force-sensor units, four corridor frames (black acrylic plates), two transparent block frames, a 45° mirror, and a CCD digital camera with the GigE interface. The force-sensor units could be easily placed in the corridor frames in combination for various tasks (usually in the center of the corridor). The two transparent block frames were adjustable to guide the rats to make a straight walk (**a**). Every force-sensor unit was composed of two acrylic plates mounted with four TBS-10 beam force sensors to measure Fx and Fy, and one steel frame mounted with two TBS-20 beam force sensors to measure Fz. The recorded maximum vertical force, Fz, which corresponded with each isolated footprint of each limb on one acrylic plate, was defined as the DWB. Meanwhile, the recorded Fx and Fy were defined as the forward (negative value)/backward (positive value) shearing force and outward (positive value)/inward (negative value) shearing force, respectively (**b**). Using this customized setup and the 45° mirror that reveals the bottom-up images, the contact points of all four limbs during walking were clearly recorded in the sagittal and coronal planes (**c**).

The kinetic analysis system included a customized corridor, a CCD digital camera with the GigE interface (acA 640–120gc; Basler Vision Technologies Inc., Jhubei City, Hsinchu County, Taiwan), and a record system. The corridor consisted of four corridor frames, four force-sensor units, two transparent block frames, and a 45° mirror (Fig. 5a). Each force plate included two force-sensor which could record three axial forces (F_x_, F_y_, and F_z_). Each force-sensor unit included six beam force-sensors (TBS-10/TBS-20; Transducer Techniques, Temecula, CA, USA), two acrylic plates, and an acrylic frame. The acrylic frame was used to mount the two TBS-20s, and thus to measure vertical force, F_z_. Four TBS-10s were mounted at the side of two acrylic plates, to measure shear force, F_x_ and F_y_ (Fig. 5b). The other four corridor frames were black acrylic plates. The force plates and frames could be easily set up in a walkway with different combinations for various tasks. The two transparent block frames were adjustable to guide rats on a straight walk. A 45°mirror was set under the force plate to reveal its bottom-up images of the force plate, which allowed the contact points of all four limbs during walking to be clearly recorded in the sagittal and coronal planes (Fig. 5c).

### Validation of customized corridor data

To test the validity of the force-sensor units, a height-adjustable bearing system was designed to test the horizontal force. A ball bearing was attached to a height gauge (No. 192–106; Mitutoyo Taiwan Co., Ltd., Taipei) to adjust the direction of the calibrated reference force to facilitate the following testing. Different reference loads (50 g, 100 g, 150 g, and 200 g for the *x* and *y* axes; 100 g, 200 g, 300 g, 400 g, and 500 g for the *z* axis) were applied to the force-sensor three times and the values measured on the force plate were recorded. For tests on the *x* and *y* axes, the height-adjustable bearing system and a fishing line connected to an s-hook were used to apply the loading (Supplementary Fig. 1). For tests on the *z* axis, the reference load was placed directly on the force plate.

### Kinetic analysis in the customized corridor

Two weeks before collagenase injections, the rats were trained three times a week on the customized corridor. All rats were made to complete at least three uninterrupted walks on the training day^20^. The unable to complete the training protocol were not included in the following experiments. On the evaluation day, a satisfactory walk was defined as an uninterrupted walk across the walkway that left ≥ 1 isolated footprints on one acrylic plate for each limb. Recordings were repeated until each rat had made four satisfactory walks. The recorded maximum vertical force, Fz, that corresponded to each isolated footprint of each limb on one acryl plate was defined as the DWB. Meanwhile, the recorded Fx was defined as the forward (landing, negative value)/backward (push-off, positive value) shearing force, an dthe recorded Fy was defined as the outward (positive value)/inward (negative value) shearing force.

### Static weight bearing paradigm

Weight distribution to the right and left hindlimbs was measured using an incapacitance tester (Singa Technology Corporation, Taipei, Taiwan) as previously reported^8^. Rats were placed in the testing box and adapted to the apparatus for approximately 5 min. The right hindlimb of the rat was positioned on the right force plate in the box, and the left hindlimb on the left one. The weight (g) distributed to each hindlimb was measured for 5 s. The data are reported as the weight of the left hindlimb as a percentage of the total weight borne by both limbs (i.e., [left hindlimb weight]/[total weight of both hindlimbs] × 100%).

### Statistical analysis

All quantitative data are expressed as means ± standard deviation (SD). The differences in histopathological grades, ultrasound feature scores, and SWBR between groups at each time point were analyzed using an independent *t*-test. The differences in the kinetic parameters between both hindlimbs or both forelimbs were evaluated using a paired *t*-test. The differences in the parameters at various time points in the same group were determined using ANOVA and post hoc Dunnett’s tests. Regression analysis was used to determine the relationship between the force plates and the calibrated reference force from the validation tests. The intraclass correlation coefficient (ICC) was used to test the repeatability of the validation tests from the force plates. The ICC was calculated using variance estimates obtained through analysis of variance. Significance was set at *p* < 0.05. Data were analyzed using SPSS 16.0 for Windows.

## Electronic supplementary material

Supplementary Information
