# Supplementary material for: Dynamic weight bearing analysis is effective for evaluation of tendinopathy using a customized corridor with multi-directional force sensors in a rat model
Source: Sci Rep. 2017 Aug 18;7:8708. doi: 10.1038/s41598-017-07812-6 (PMC5562883; doi:10.1038/s41598-017-07812-6)
Supplement: Supplementary file 1 — Supplementary Information [file 41598_2017_7812_MOESM1_ESM.pdf]

Dynamic weight bearing analysis is effective for evaluation of tendinopathy using a customized corridor with multi-directional force sensors in a rat model

Po-Ting Wu[1,2,3,4], Chieh-Hsiang Hsu[3], Fong-Chin Su[3,4], I-Ming Jou[5,1,2], Shih-Yao Chen[6], Chao-Liang Wu[7], Wei-Ren Su[1,2], Li-ChiehKuo[8]

[1]Department of Orthopedics, College of Medicine, National Cheng Kung University, Tainan, Taiwan

[2]Department of Orthopedics, National Cheng Kung University Hospital, College of Medicine, National Cheng Kung University, Tainan, Taiwan

[3]Department of Biomedical Engineering, National Cheng Kung University, Tainan, Taiwan

[4]Medical Device Innovation Center, National Cheng Kung University, Tainan, Taiwan

[5]Department of Orthopedics, E-DA Hospital, Kaohsiung, Taiwan

[6]Department of Internal Medicine, College of Medicine, National Cheng Kung University, Tainan, Taiwan

[7]Department of Biochemistry and Molecular Biology, College of Medicine, National Cheng Kung University, Tainan, Taiwan

[8]Department of Occupational Therapy, College of Medicine, National Cheng Kung University, Tainan, Taiwan

Supplementary Table 1 The ICC values and R^2 values from the linear regression analyses of force plates in all three axes on the customized corridor.

Force plate	ICC Test			R^2		
	Fz	Fy	Fx	Fz	Fy	Fx
No.1	1.00	1.00	1.00	1.0000	1.0000	0.9995
No.2	1.00	1.00	1.00	0.9990	1.0000	0.9984
No.3	1.00	1.00	1.00	0.9990	1.0000	0.9998
No.4	1.00	1.00	1.00	1.000	1.0000	1.0000

ICC, intraclass correlation coefficient

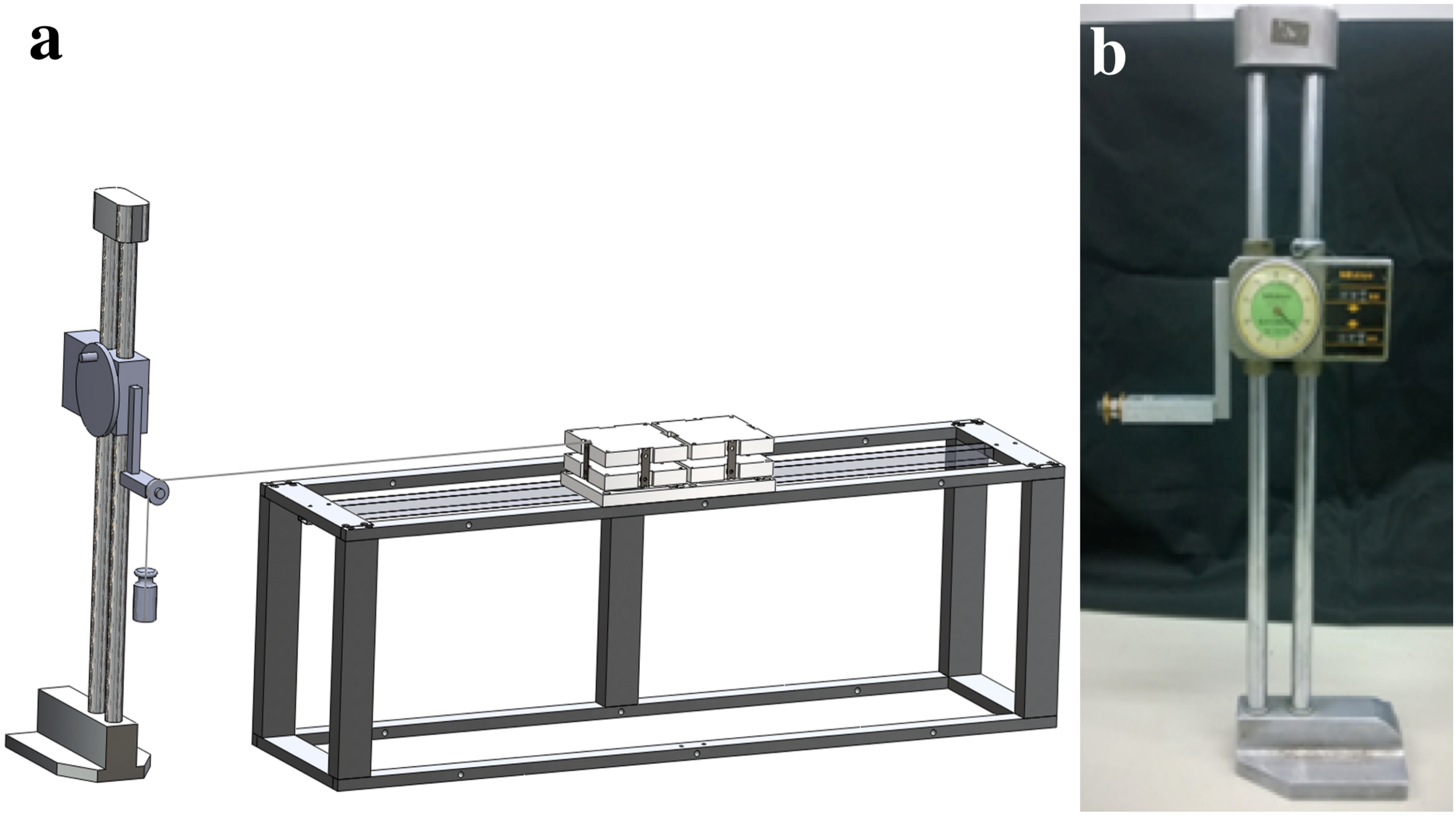
a
b

Supplementary figure legends

Supplementary Figure 1. Illustration of the height-adjustable bearing system for validation of force plates (a) and the calibrated reference load applied for validation of force plate on the x and y axes (b).
